# Segment-Targeted Resection Strategy for Bilateral Hepatolithiasis: A Retrospective Study in Bangladesh

**DOI:** 10.7759/cureus.117942

**Published:** 2026-10-14

**Authors:** Bidhan C Das, Amit Chowdhury, Anupam Debnath, MD Mamun, Gopal Chandra Pal, Dibakor D Roy, Sabuj Kumar Patra

**Affiliations:** 1 Department of Hepatobiliary, Pancreatic, and Liver Transplant Surgery, Bangladesh Medical University, Dhaka, BGD; 2 Department of Hepatobiliary Surgery, United Medical College Hospital, Dhaka, BGD; 3 Department of Hepatobiliary Surgery, Evercare Hospital Dhaka, Dhaka, BGD; 4 Department of Surgery, Directorate General of Health Services, Dhaka, BGD

**Keywords:** bilateral hepatolithiasis, cholangitis, hepaticojejunostomy, hepatic resection, intrahepatic stones, segmentectomy, surgical technique

## Abstract

Background: Bilateral hepatolithiasis is difficult to treat because stones may be distributed across multiple intrahepatic ducts and may persist despite duct exploration and biliary drainage. We evaluated a segment-targeted surgical strategy designed to remove the hepatic segments most frequently involved in our patients with bilateral hepatolithiasis.

Materials and methods: We retrospectively reviewed 120 patients who underwent surgery for hepatolithiasis at Bangladesh Medical University, Dhaka, between 2010 and 2024. Forty-six patients had bilateral hepatolithiasis on preoperative magnetic resonance cholangiopancreatography, of whom 40 with available follow-up data were included. Patients treated from 2010 to 2020 formed Group I (n = 26) and underwent conventional surgical management consisting of extended choledocholithotomy and/or extrahepatic bile duct excision with biliary reconstruction. Patients treated from 2021 to 2024 formed Group II (n = 14) and underwent combined resection of segments 2, 3, 5, and 6, extrahepatic bile duct excision, irrigation of the remaining intrahepatic ducts, and Roux-en-Y hepaticojejunostomy. Complete stone clearance, residual stones, recurrent postoperative cholangitis, hospital readmission for cholangitis, and postoperative endoscopic retrograde cholangiopancreatography (ERCP) were compared.

Results: Complete stone clearance was documented in 12 of 26 patients (46.2%) in Group I and 14 of 14 patients (100%) in Group II (Fisher's exact test, p < 0.001). Residual stones were identified in 14 Group I patients (53.8%) and none in Group II. Recurrent postoperative cholangitis requiring hospital readmission occurred in 14 Group I patients (53.8%) and none in Group II. Six Group I patients (23.1%) underwent postoperative ERCP for stone or stent management; no Group II patient required this intervention. Follow-up duration was up to 14 years in Group I and ranged from four months to four years in Group II.

Conclusions: In this single-center retrospective cohort, segment-targeted resection combined with extrahepatic bile duct excision and Roux-en-Y hepaticojejunostomy was associated with complete stone clearance and no documented recurrent postoperative cholangitis during follow-up. These findings support prospective evaluation of the technique in larger cohorts with standardized follow-up.

## Introduction

Hepatolithiasis refers to stones within the intrahepatic bile ducts proximal to the hepatic hilum and may coexist with extrahepatic biliary stones. The disease is particularly prevalent in East and Southeast Asia and is associated with biliary stasis, infection, abnormal ductal anatomy, and other local and systemic factors [1-7]. Persistent stones and biliary strictures may lead to recurrent cholangitis, secondary biliary cirrhosis, and, in some patients, an increased risk of intrahepatic cholangiocarcinoma [4,5].

Management aims to achieve complete stone clearance, relieve biliary obstruction, correct relevant strictures, and prevent recurrence. Available approaches include endoscopic or percutaneous stone extraction, biliary drainage, and hepatic resection. Anatomical hepatic resection is particularly useful when disease is localized to specific hepatic segments or associated with segmental atrophy, although the management of residual or bilateral disease remains challenging [8-15].

In our surgical practice, preoperative magnetic resonance cholangiopancreatography (MRCP) suggested a recurrent stone distribution involving the left lateral segments and selected right-sided segments. This observation led to a segment-targeted surgical approach consisting of resection of segments 2, 3, 5, and 6; excision of the extrahepatic bile duct; irrigation of the remaining intrahepatic ducts; and Roux-en-Y hepaticojejunostomy. This study describes the technique and compares postoperative stone clearance and clinical outcomes with those of patients treated with our earlier conventional surgical approach.

## Materials and methods

Study design and setting

This retrospective cohort study was conducted in the Department of Hepatobiliary, Pancreatic, and Liver Transplant Surgery at Bangladesh Medical University, Dhaka, Bangladesh. Medical records of 120 patients who underwent surgery for hepatolithiasis between 2010 and 2024 were reviewed. Bilateral hepatolithiasis was defined clinically and radiologically by the presence of intrahepatic stones involving both sides of the liver on preoperative MRCP. The study included consecutive adult patients with MRCP-confirmed bilateral hepatolithiasis. The exclusion criteria included incomplete medical records, unavailable follow-up data (n = 6), and end-stage decompensated liver disease that rendered surgical resection unsafe.

Of the 120 surgically treated patients, 46 (38.3%) had bilateral hepatolithiasis. Six patients were excluded from the final analysis because adequate follow-up data were unavailable, leaving 40 patients for analysis. Patients were divided according to the surgical strategy used during two consecutive periods. This historical allocation was determined by the evolution of the surgical technique rather than randomization.

Surgical groups

All surgical procedures in both groups were performed or directly supervised by senior hepatopancreatobiliary surgeons to ensure technical consistency across the cohort. Group I (2010-2020; n = 26), the traditional approach, underwent conventional surgical management. Depending on the anatomical findings, procedures included extended choledocholithotomy and/or extrahepatic bile duct excision. Intrahepatic ducts were irrigated, and biliary continuity was restored with Roux-en-Y hepaticojejunostomy.

Group II (2021-2024; n = 14), the new approach, underwent the new segment-targeted procedure. The operation consisted of combined resection of hepatic segments 2, 3, 5, and 6 followed by excision of the extrahepatic bile duct. The remaining intrahepatic ducts were irrigated with normal saline, after which biliary reconstruction was performed using Roux-en-Y hepaticojejunostomy. Segment-targeted resection was indicated for patients whose preoperative MRCP showed predominant stone involvement in the left lateral segments (S2 and S3) and selected right-sided segments (S5 and S6), provided adequate functional liver parenchyma could be preserved. The intended rationale was to remove the segments most consistently affected by stones while preserving the remaining functional hepatic parenchyma.

Standard perioperative care included prophylactic broad-spectrum antibiotics, intraoperative hemodynamic and fluid management, and daily postoperative monitoring of liver biochemical parameters, serum bilirubin levels, and drain output.

Preoperative assessment and follow-up

Stone distribution was mapped using preoperative MRCP. Residual stones, biliary architecture, and complete stone clearance were assessed using postoperative MRCP at approximately one to three months after surgery in all patients. Patients underwent follow-up evaluations at one- to three-month intervals during the first postoperative year. Data on postoperative cholangitis, hospital readmission, and endoscopic retrograde cholangiopancreatography (ERCP), with or without stone extraction or stent placement, were extracted from the available medical records.

Outcomes

The primary outcome was complete stone clearance after the index operation, defined as the absence of intrahepatic or extrahepatic stones on postoperative MRCP. Secondary outcomes included residual stones, recurrent postoperative cholangitis, hospital readmission for cholangitis, and postoperative ERCP for stone or stent management. Recurrent postoperative cholangitis was defined as an episode of biliary infection presenting with fever, right upper quadrant pain, and jaundice that required hospital readmission for intravenous antibiotic administration or ERCP/stent intervention. Other outcomes included residual stones, postoperative cholangitis requiring hospital-based treatment, hospital readmission, and postoperative ERCP for stone or stent management.

Ethical approval

The Institutional Review Board of Bangladesh Medical University approved the study protocol (approval number: PR-1349). The study used retrospective clinical records.

Statistical analysis

Data were analyzed using SPSS Statistics version 22.0 (IBM Corp. Released 2013. IBM SPSS Statistics for Windows, Version 22.0. Armonk, NY). Continuous variables were summarized as mean ± standard deviation when appropriate. Categorical variables were summarized as frequencies and percentages. Categorical outcomes were compared using Fisher's exact test, as expected cell counts were small. For the principal comparison of complete stone clearance, Fisher's exact test yielded p < 0.001. A two-sided p-value of less than 0.05 was considered statistically significant.

## Results

Demographics

Group I included 10 men and 16 women, with a mean age of 37 ± 12 years. Group II included five men and nine women, with a mean age of 34 ± 6.2 years. In both groups, stones were most frequently identified in segments 2, 3, and 6. Extrahepatic stones were commonly identified in the hilar region, common bile duct, and gallbladder. Table 1 shows the distribution of stones by anatomical location.

**Table 1:** Distribution of intrahepatic and extrahepatic stones in the study cohort Percentages are calculated within each group.

Anatomical location	Group I (n = 26)	Group II (n = 14)
Intrahepatic segments		
Segment 1	0 (0%)	0 (0%)
Segment 2	24 (92.3%)	13 (92.9%)
Segment 3	24 (92.3%)	14 (100%)
Segment 4	0 (0%)	2 (14.3%)
Segment 5	6 (23.1%)	2 (14.3%)
Segment 6	18 (69.2%)	12 (85.7%)
Segment 7	2 (7.7%)	2 (14.3%)
Segment 8	1 (3.8%)	0 (0%)
Extrahepatic biliary tree		
Hilar area	13 (50.0%)	8 (57.1%)
Common bile duct	22 (84.6%)	13 (92.9%)
Gallbladder	23 (88.5%)	14 (100%)

Postoperative outcomes

Complete stone clearance was achieved in 12 of 26 patients (46.2%) in Group I and in all 14 patients (100%) in Group II. The difference was statistically significant (Fisher's exact test, p < 0.001). Residual stones were documented in 14 Group I patients (53.8%) compared with none in Group II.

Fourteen patients in Group I (53.8%) experienced recurrent postoperative cholangitis requiring hospital readmission. Of these, six underwent postoperative ERCP to manage residual extrahepatic stones and/or biliary stents, while eight received conservative treatment with antibiotics and analgesics, according to the available records. No patient in Group II developed documented recurrent postoperative cholangitis, required hospital readmission for cholangitis, or underwent postoperative ERCP during the available follow-up period (Table 2).

**Table 2:** Comparison of postoperative outcomes between the two surgical groups * Fisher's exact test ERCP: endoscopic retrograde cholangiopancreatography

Clinical outcome	Group I (n = 26) (traditional approach)	Group II (n = 14) (new approach)	p-value
Complete stone clearance	12 (46.2%)	14 (100%)	<0.001*
Residual stones	14 (53.8%)	0 (0%)	
Recurrent postoperative cholangitis	14 (53.8%)	0 (0%)	
Hospital readmission for cholangitis	14 (53.8%)	0 (0%)	
Postoperative ERCP for stone or stent management	6 (23.1%)	0 (0%)	

Follow-up duration differed between the groups because of the historical study design. Group I patients had up to 14 years of follow-up, whereas Group II patients had four months to four years of follow-up. Therefore, the absence of recurrence events in Group II should not be interpreted as proof of longer-term superiority.

## Discussion

Anatomical hepatic resection is a definitive surgical treatment option for appropriately selected patients with hepatolithiasis. Although highly effective for unilobar disease, complete stone clearance is significantly more challenging when stones involve multiple hepatic segments bilaterally [13]. The literature reports high residual stone and recurrence rates after conventional biliary drainage or minimally invasive procedures. For instance, Otani et al. reported a 31.5% recurrence rate within five years with repeated percutaneous transhepatic cholangioscopic lithotomy, whereas our cohort had a 53.8% residual stone rate after extended choledochotomy [16].

The principal challenge in bilateral hepatolithiasis is achieving durable clearance when stones are distributed across multiple intrahepatic ducts. Surgical treatment is generally favored when disease is anatomically localized, when segmental hepatic atrophy is present, or when strictures and recurrent infection make repeated nonoperative stone extraction less satisfactory [8-15]. Previous reports show that hepatic resection can provide durable clearance in selected patients, while residual stones remain a major cause of recurrent cholangitis after non-resectional treatment [14-16].

Our findings indicate that intrahepatic stones were predominantly distributed in the left lateral segments (S2 and S3) and right posterior segment (S6). This distribution is consistent with findings reported by Li et al. [17]. This pattern may reflect the anatomy of the biliary tree: the ducts draining the lateral and posterior segments may be relatively long and join larger bile ducts at acute angles. This configuration may impede gravity-driven bile flow, increasing the likelihood of bile stasis and subsequent lithogenesis. Conversely, the ducts of segments S4, S7, and S8 are shorter and join at obtuse angles, facilitating gravity-assisted drainage and reducing the risk of stone formation. The underlying concept is that removing repeatedly affected ductal territories may reduce the burden of diseased intrahepatic ducts and improve the likelihood of complete stone clearance.

In our cohort, complete stone clearance rates were 46.2% with the conventional surgical approach and 100% with the segment-targeted surgical approach. Residual stones and recurrent postoperative cholangitis were each documented in 53.8% of Group I patients but were not documented in Group II during the available follow-up. These findings are clinically encouraging and consistent with the broader surgical literature supporting anatomical hepatic resection in appropriately selected patients with hepatolithiasis [13-17].

However, the proposed mechanism should be interpreted cautiously. The observed stone distribution may be attributable to differences in ductal angulation and gravity-assisted drainage. Although biliary anatomy and impaired drainage plausibly contribute to stone formation, the present study did not directly measure ductal angles, bile flow, intraductal pressure, or other physiological variables. Accordingly, these anatomical explanations should be considered hypotheses generated by the observed distribution rather than demonstrated causal mechanisms.

The study has several important limitations. First, it is retrospective and single-center, with only 40 evaluable patients. Second, treatment allocation was chronological rather than randomized, creating potential confounding from changes in surgical expertise, perioperative care, imaging, and patient selection over time. Third, follow-up duration was substantially longer in Group I than in Group II, making direct comparisons of late recurrence and long-term cholangitis rates inappropriate. Fourth, the available dataset lacks detailed information on operative time, blood loss, postoperative morbidity, liver function, bile leakage, strictures, mortality, quality of life, or stone composition. Fifth, six patients with bilateral hepatolithiasis were lost to follow-up, introducing potential attrition bias. These limitations mean the findings should be viewed as preliminary.

Despite these limitations, the study provides a clinically testable operative concept for bilateral hepatolithiasis. A prospective study using standardized inclusion criteria, uniform postoperative imaging, predefined definitions of stone clearance and recurrent cholangitis, and comparable follow-up duration would be appropriate to determine whether the apparent benefit of the segment-targeted surgical approach persists beyond the early postoperative period.

## Conclusions

In this retrospective, single-center series, combined resection of segments 2, 3, 5, and 6 with extrahepatic bile duct excision, irrigation of the remaining intrahepatic ducts, and Roux-en-Y hepaticojejunostomy was associated with complete stone clearance in all patients treated with the segment-targeted surgical approach.

The findings are encouraging but should not yet be considered definitive evidence of superiority. Larger prospective cohorts with standardized follow-up are needed to evaluate the reproducibility, safety, and long-term durability of this approach and to establish appropriate patient selection criteria for this segment-targeted surgical strategy.
